# Influence of state anxiety and trate anxiety in postoperative in oral surgery

**DOI:** 10.4317/medoral.19604

**Published:** 2014-03-08

**Authors:** Daniel Torres-Lagares, Concha Recio-Lora, Gabriel Castillo-Dalí, Gonzalo Ruiz-de-León-Hernández, Pilar Hita-Iglesias, Maria A. Serrera-Figallo, Juan J. Segura-Egea, José L Gutiérrez-Pérez

**Affiliations:** 1 Master in Oral Surgery. University of Seville, Spain

## Abstract

Introduction: The aim of this article was to study the influence of anxiety (both state and trait) in postoperative recovery after extraction of third molar together, to establish the role of each of the aspects of anxiety in the results you obtained in an independent and complementary way. Material and Methods: We performed a prospective study of a consecutive series of 88 patients who underwent lower third molar extractions. Before being provided with any information about the operation, patients were asked to complete the Spielberger State-Trait Anxiety Inventory-Trait and State. We have evaluated postoperative swelling and pain, patients completed a 10-point visual analog scale (VAS) at home each day (at approximately the same time of day as the operation) until day 8 after surgery, when the sutures were removed. Results: Regarding postoperative variables between positive and negative trait anxiety groups, consumption of analgesic drugs was higher in positive trait anxiety group in a statistically significant way, while these differences were detected only on specific occasions regarding pain and swelling. Discussion: In the present study, anxiety was taken into account and showed a significant effect in explaining postoperative pain and taking analgesics.

** Key words:**Anxiety, satisfaction, third molar surgery, Spielberger state-trait anxiety inventory.

## Introduction

Anxiety is an emotional reaction defined as tension (stress), apprehension, nervousness and concerns caused by an intangible and diffuse advancing threat or approaching danger, accompanied by activation of the autonomous nervous system (1).

Moderate to severe acute postoperative pain occurs frequently after different surgical procedures and involves up to 50% of hospitalized patients and 40% of patients undergoing ambulatory surgery (2).

An aspect that is far from completely clear is the wide variation in patients’ experiences of pain after similar types of surgical injury (3).

Numerous authors studied the influence of anxiety in the experience of pain (measured in an objective as well as in a subjective way) suffered by the patient during and after the surgery (2,3). The fact that individual put face to surgery with high levels of anxiety may have negative influences in postsurgical physical and mental recovery, such as long hospital stays or greater need for analgesics, which means a harmful both to the individual and the health system by its high cost (4,5).

Anxiety has been studied in two ways: as a personality trait (in which case we refer to it as trait anxiety) and as state of the person (in which case we refer to it state anxiety).

Trait anxiety is a relatively stable tendency toward the kind of anxiety that anyone can suffer when facing situations perceived as threatening. (1) State anxiety is felt as a transitory emotional condition of the human body, characterized by subjective and consciously perceived strain and apprehension feelings and by hyperactivity of the autonomic nervous system. State anxiety also includes dental anxiety, an anxious state in a patient caused by dental treatment (1).

Both variables are, in principle, independent. That means that a person with trait anxiety may not present a state anxiety at one time and a person without this trait in his personalit can manifest anxiety. The influence of both variables related to anxiety, trait and state, regarding postoperative variables, has been approached in several studies (6).

However, these studies have not been observed in a complementary but isolated way, and using different scales that make it difficult to compare results (6-8).

The aim of this article is to study the influence of anxiety (both state and trait) in postoperative recovery after extraction of third molar together, to establish the role of each of the aspects of anxiety in the results you obtained in an independent and complementary way.

## Material and Methods

- Patient selection

Between January 2009 and July 2009, we performed a prospective study of a consecutive series of 88 patients who underwent lower third molar extractions. We excluded patients who did not comply with the formal requirements of the study (exclusion criteria: patients that have undergone further oral surgery; patients that don´t consent to participate; patients with impaired cognitive and communication abilities; patients with history of anxiety attacks and anxiolytic treatment; patients which questionnaires have errors; patients which lower third molar extraction lasted more than 30 minutes (because with this duration, we applied a pharmacological treatment different (corticoids). The sample was composed of 88 patients undergoing a lower third molar removal for the first time (43 left and 45 right), 57 (64,8%) of whom were women and 31 (35,2%) men.

Patient mean age was 30,4 years ± 10,1. All patients were healthy, with no serious medical conditions or blood dyscrasia. None of the patients had acute pericoronitis or severe periodontal disease at the time of surgery.

- Anxiety evaluation

Before being provided with any information about the operation (60 minutes before), patients were asked to complete the Spielberger State-Trait Anxiety Inventory- Trait (STAI-T) (1). This 20-item self-evaluation questionnaire is scored using a 4-level frequency scale, ranging from “almost never” to “almost always,” reflecting different degrees of anxiety about situations that subjects perceive as threatening. The patients were subsequently informed about the surgery and postoperative recovery.

Before entering the treatment room (30 minutes before), patients, by themselves and in a quiet “non-dental” room, filled out the Spielberger State-Trait Anxiety Inventory- State (STAI-S) (1). The STAI-S (1) is a 20-item self-evaluation questionnaire, scored using a 4-level frequency scale ranging from 0 to 3, that assesses transient emotional state or condition as characterized by subjective feelings of tension and apprehension that can fluctuate in time and intensity. Social profile data were also taken (profession, age, sex y marital status).

The positive level for STAI-T and STAI-S was, from 0 to 60, of 20 for the young men (percentile 50 in studies of validation of the above mentioned questionnaire) and of 22 for young women (percentile 50 in studies of validation of the above mentioned questionnaire).

- Surgical Procedure 

All interventions were performed by postgraduate students at the University of Seville (Spain) with the same training level. A total of eight surgeons made the surgical treatments. Surgery was in all cases performed under local nerve-block anesthesia of the inferior dental nerve, lingual nerve, and buccal nerve with 2 x 1.8-ml capsules of 4% articaine with 1:200,000 epinephrine (Articaine; Inibsa, Barcelona, Spain). A mucoperiosteal flap was raised by incision distal to the lower second molar along the length of the anterior border of the ascending ramus of the mandible, with another incision mesial to the same molar. Osteotomy, coronal section, or root section was then performed as required, and the wound was closed with 3/0 silk. A piece of folded gauze was applied to the wound to aid hemostasis.

All patients received an antibiotic (amoxicillin 500 mg/8 h for 7 days, starting the day before surgery), and an antiinflammatory/analgesic agent (ibuprofen 600 mg on demand, with a maximum of 8 tablets per day, for 7 days starting after surgery). Patients were also given appropriate instructions and recommendations regarding the postoperative recovery period. The sutures were removed 1 week later.

- Evaluation of postoperative 

To evaluate postoperative swelling and pain, patients completed a 10-point visual analog scale (VAS) of 10 mm of length (marking at the beginning “no pain” or “no swelling”, and at the end “the worst pain possible” or “the worst swelling possible”) (9) at home each day (at approximately the same time of day as the operation) until day 8 after surgery, when the sutures were removed. Patients were also informed of the need of record the analgesic medication taken during the different days of the study.

- Statistical analysis 

Data collected were entered into an MS-Excel data table (Microsoft Corp. – EE.UU.) and exported to SPSS for Windows v.11 (SPSS Inc. – EE.UU.). Normality of data were confirmed by Kolmogorov-Smirnov test, comparing the averages of the different study groups by the t of Student and ANOVA tests with later Bonferroni test. Later, statistical power analysis was made.

## Results

Our study included 88 patients. 30 of them presented anxiety as personality trait (37,5%) and 41 presented anxiety state in the study (45,4%).

Regarding postoperative variables between positive and negative trait anxiety groups ( Table 1), we can see that consumption of analgesic drugs was higher in positive trait anxiety group in a statistically significant way, almost every day of the study, while these differences were detected only on specific occasions regarding pain (at the end of the study period) and swelling (at the beginning of the study period).

In relation to the difference between the studied variables regarding the positive trait anxiety compared to the negative group ( Table 2), no differences were found in relation to the consumption of drugs, while statistically significant differences were found in relation to pain during the whole study period, ant to swelling at the beginning of it.

**Table 1:**
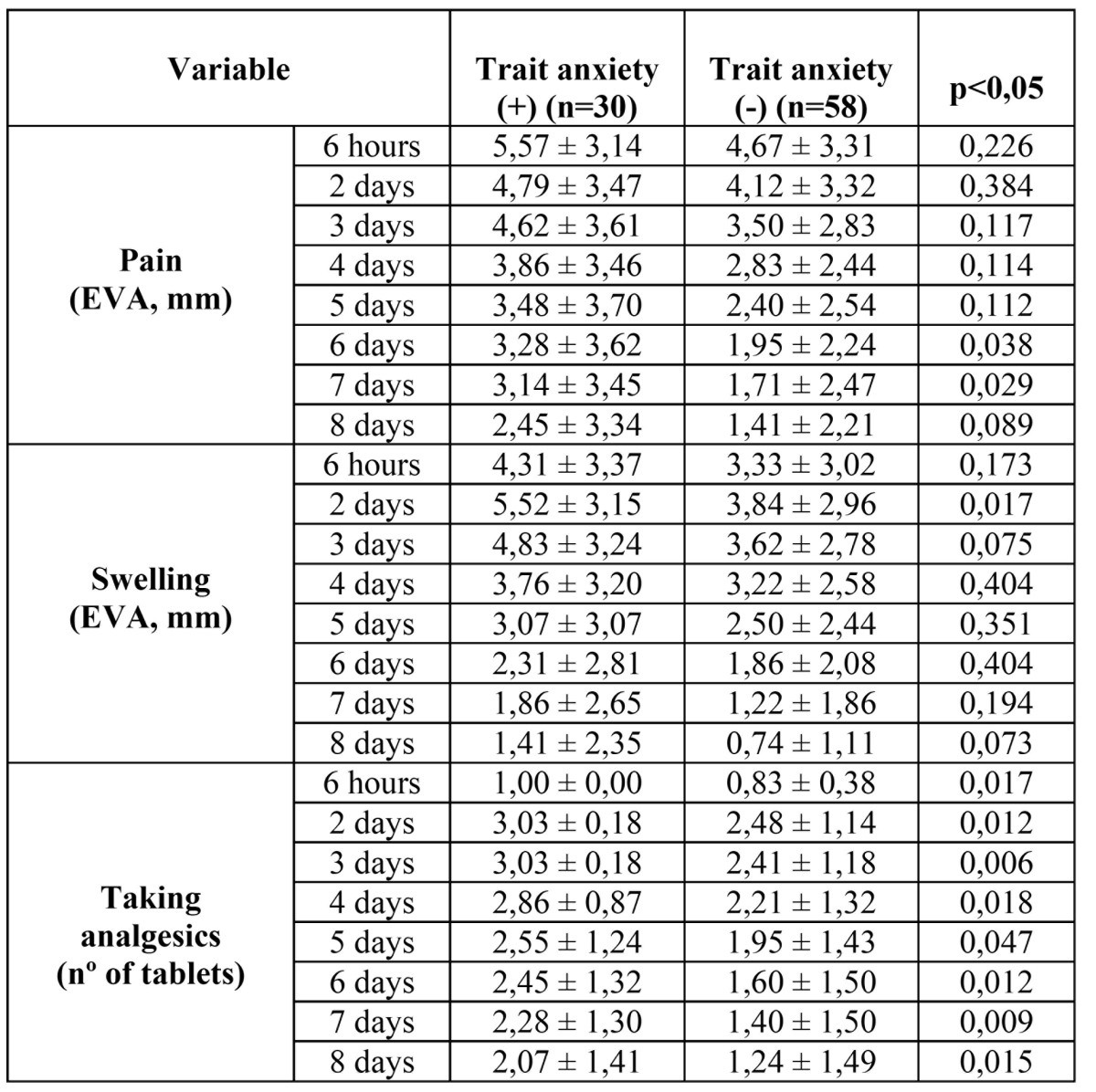
Data concerning the assessment of pain, swelling and pain medication as making the appearance of trait anxiety in patients in the sample.

**Table 2:**
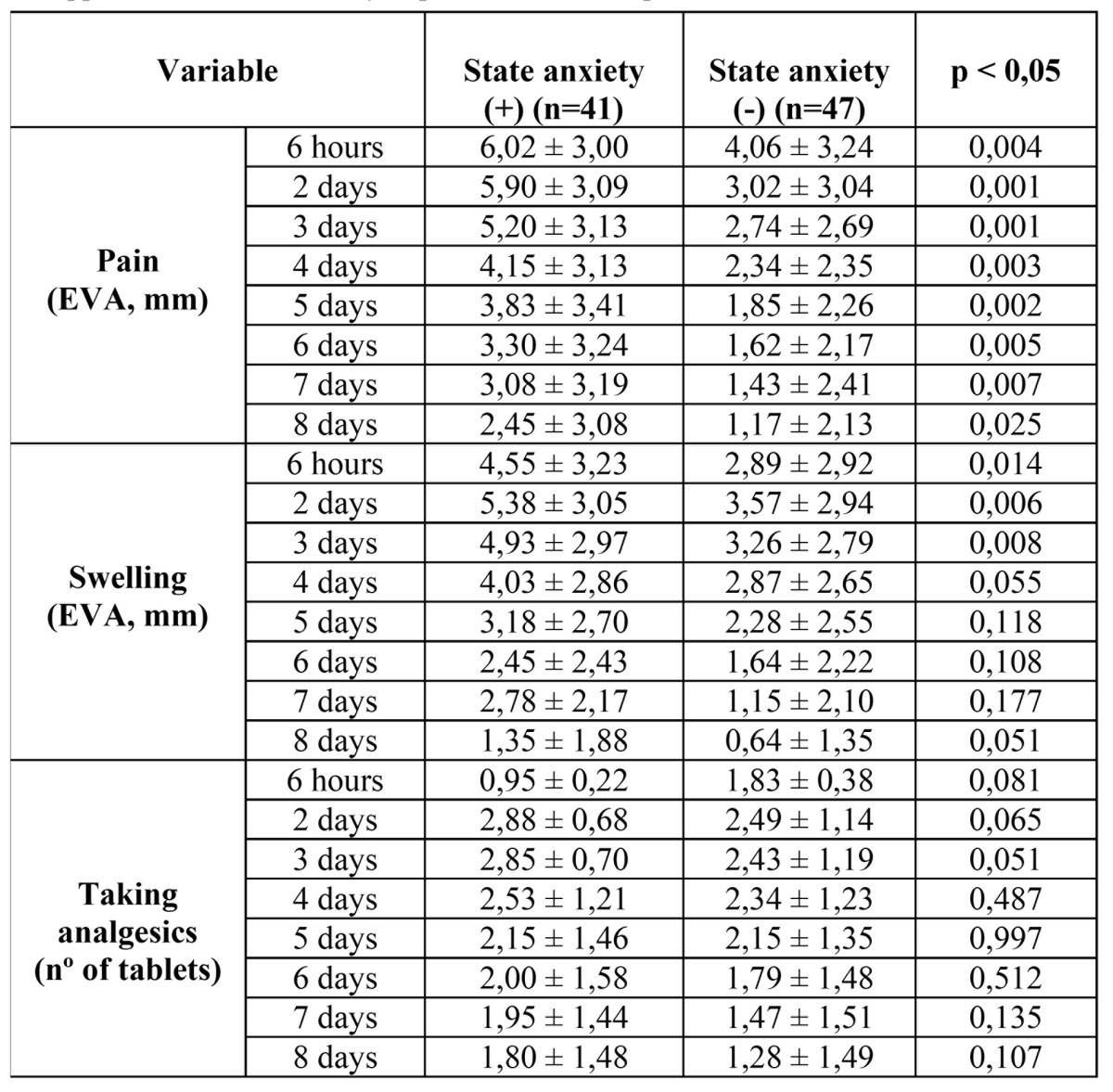
Data concerning the assessment of pain, swelling and pain medication as making the appearance of state anxiety in patients in the sample.

Crossing both variables (state anxiety (SA) and trait anxiety (TA)) we founded that 20 patients presented positive SA (+) and negative TA (-) (22,72%), 9 patients presented SA- and T+ (10,22%), 21 patients presented SA+ and TA+ (23,86%), and 38 patients presented SA- and TA- (43,18%) ( Table 3).

**Table 3:**
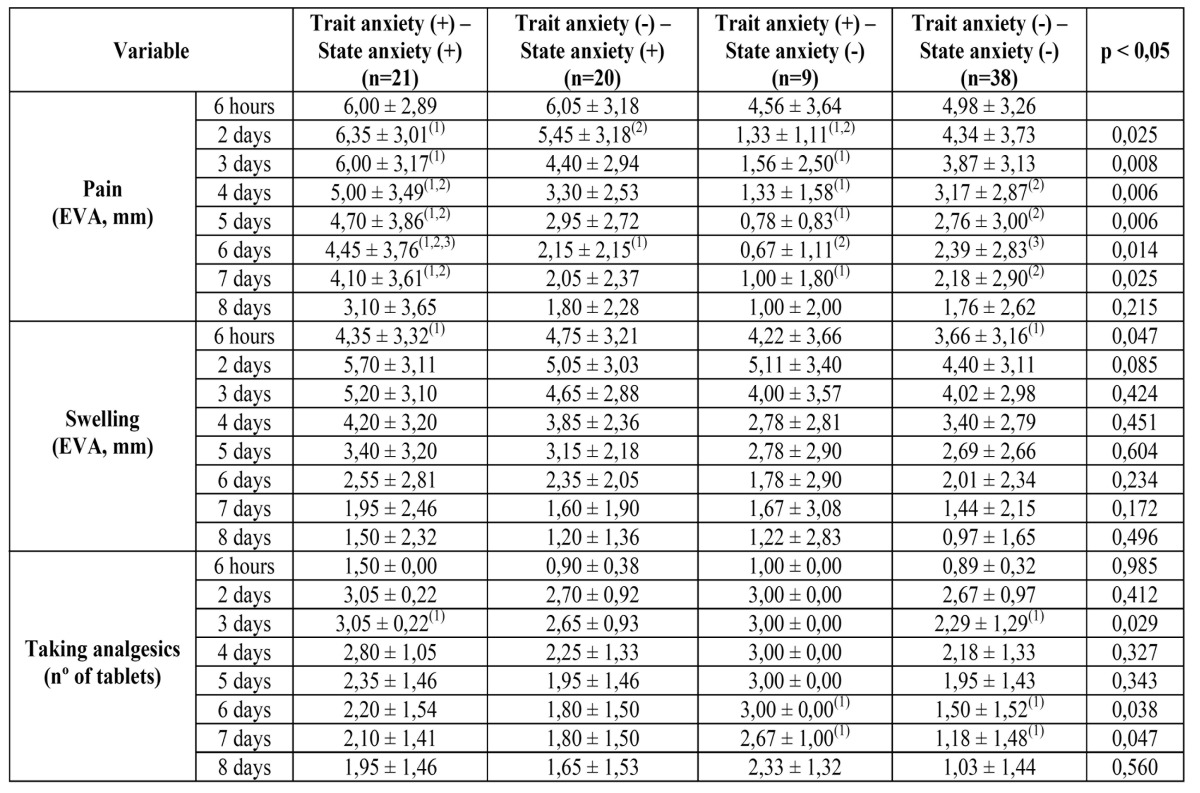
Data concerning the assessment of pain, swelling and pain medication as making the appearance of state anxiety or trait anxiety in patients in the sample. (The superscripts indicate, in the same row, the data pairs with statistically significant differences in the Bonferroni test (*p*<0,005).

Edema did not show statistically significant values among the four groups. Taking drugs was higher in TA+SA- group tan in TA-SA- group in the last days of the study.

Perceived pain was higher in TA+SA+ than in the rest of groups during all days of the study, and these differences were statistically significant from the second to the seventh day of the study.

Regarding the distribution of positive trait anxiety compared to positive state anxiety ( Table 4), patients with positive trait anxiety presented positive state anxiety in a 70% (21 out of 30) while in patients with negative trait anxiety this percentage was almost half (34,48%; 20 out of 58). This difference was statistically significant.

Statistical power

**Table 4:**
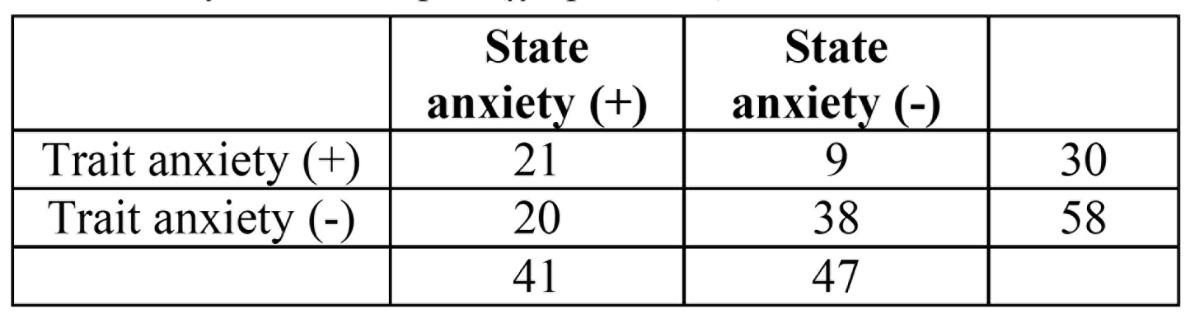
Relationship of the presentation of anxiety as state and trait anxiety in the sample. (χ2, *p*=0,002).

We have made the statistical power analysis in all the comparisons. For the first test ( Table 1) (30 and 61 patients, alpha = 0,05 and beta = 0,2 (power 80 %), it might detect a difference of 1,2 mm in EVA, which is clinical relevant. For the second test ( Table 2), it might detect a difference of 1 mm in EVA with the same characteristics.

In the test presented in Table 3, this one would have a power of 80 % to detect changes of 2 mm in the EVA. To detect a difference of 1 mm in EVA the power of the test war only 30 %. For what the not significant differences established in the above mentioned table must not think like conclusively.

## Discussion

Liddell and Locker, in their study, found that preoperative anxiety decreased with age (10). Hägglin *et al*. (11) explained that technological advances in dentistry can decrease this anxiety. Liau *et al*. (12), found that younger patients had higher anxiety, arguing that experience and familiarity are important factors.

In the present study, anxiety was taken into account and showed a significant effect in explaining postoperative pain (state anxiety) and taking analgesics (trait anxiety), however, it fails to point out differences in the edema felt by patients beyond the first few days of the study (state of anxiety). Although relations between negative feeling and pain-related unpleasantness have been found in several studies (7). Taenzer *et al*. (13) subsequently confirmed that high levels of trait anxiety meant an increased perception of pain, and this has also been confirmed for other types of surgeries (14,15).

Our results are coherent with González-Lemmonier *et al*. (7) and Vassend (16), and these findings are consistent with those obtained of George *et al*., (17) who concluded that high levels of trait anxiety were associated with a poorer recovery. We agree with other authors who suggest that pain overestimation and a fear of pain is manifested by people with high dental anxiety (18-20).

Is still under discussion the predictive value of anxiety about postoperative pain. While Vallerand *et al*. (21) stated even that trait anxiety was an accurate predictor of postoperative pain and oral surgery recovery other authors do not share this opinion (6).

It has been shown that psychological stress can have many physical effects, ranging from increased sympathetic-adrenergic activity to illness susceptibility. Stress may even adversely affect physical recovery after surgery. Several studies report a relationship between psychological factors and postsurgical recovery; however, the results are neither clear nor compelling (9,15,22-24).

As pointed out by Hoogenboom and Vielvoye-Kerkmeer (25), our data indicate that the use and effect of painkillers administered after third molar extraction depended on the level of anxiety. In our study, this feature is observed between the groups positive and negative trait anxiety.

It has not been developed before the relationship between trait anxiety dealing with anxiety as a state, as it has been done in our study. Our data indicate that a patient with personality trait anxiety is twice as likely to be anxious (as a state) before dental treatment than a patient without this personality trait.

In relation to the crossing of variables SA and TA, we note that the positive combination of both variables are detected, the highest values of postoperative pain, while the lowest values (even more than the values of SA and TA negative group) are detected in TA+ and SA- , indicating the increased importance of anxiety as a state in this section. However, the few statistically significant differences found relating to the consumption of painkillers are linked to anxiety trait.

The results of our study are of clinical usefulness in two ways. The first one is that the influence of the anxiety strait and trate in the postoperatory is demonstrated. Previously only the influence of the anxiety state had been demonstrated. Therefore, there should be promoted any activity that improves or diminishes the anxiety, being based on its clinical benefits, especially if they are innocuous.

Our information also would support the preoperatory administration of tranquillizers, though this should be evaluated together with the costs and possible risks of the medication.

In conclusion, and according to our data, anxiety influences pain perceived by the patient during the oral surgery postoperative and taking painkillers. However, this does not influence the perception of inflammation during the same period. It also confirms that trait and state components of anxiety influence these clinical values differently.
